# On the periphery of HIV and AIDS: Reflections on stress as experienced by caregivers in a child residential care facility in South Africa

**DOI:** 10.1080/17290376.2017.1389300

**Published:** 2017-10-24

**Authors:** Kesh Mohangi, Chereen Pretorius

**Affiliations:** ^a^ PhD, is an associate professor in the Department of Psychology of Education, University of South Africa, Pretoria, South Africa; ^b^ Department of Educational Psychology, University of Pretoria, Pretoria, South Africa

**Keywords:** *caregivers*, *coping*, *institutional care*, *orphaned and vulnerable children*, *stress*, *soignants*, *adaptation*, *soins en établissement*, *enfants orphelins et vulnérables*, *stress*

## Abstract

Few researchers have investigated how female caregivers of institutionalised children, especially those affected by HIV and AIDS, experience stress. The role played by caregivers cannot be overemphasised; yet caregivers who work in institutions caring for orphaned and/or abandoned children affected by HIV and AIDS, are often marginalised and on the periphery of the HIV and AIDS pandemic. The implication is that insufficient attention or consideration is given to the importance of the role they play in these children’s lives. The objective of the study was to explore how female caregivers of institutionalised children affected by HIV and AIDS experience stress. A qualitative research project with a case study design was conducted. The purposively selected participants from a previously identified care facility were seven females in the age ranges of 35–59. Data was gathered during individual interviews and focus group discussions. Thematic content analysis of the data yielded the following themes: (1) contextualising caregiving as ‘work’; (2) stresses linked to caregiving; and (3) coping with stress. Findings from this study indicated that participants experienced caregiving in an institution as stressful, demotivating, and emotionally burdensome. Moreover, caregivers working in an environment of HIV and AIDS experienced additional stress related to organisational and management impediments, lack of emotional and practical support, inadequate training, discipline difficulties, and lack of respect and appreciation from the children in their care. It is recommended that training and management support as well as personal support and counselling for caregivers in the institutional context could help them to cope better, feel empowered and to potentially elevate their status as valued members of society.

## Résumé

Peu de chercheurs ont étudié comment les femmes soignantes d'enfants placés en établissement, en particulier ceux qui sont affectés par le VIH et le sida, éprouvent du stress. Le rôle joué par les soignants ne peut pas être surestimé; mais les soignants qui travaillent dans des institutions s'occupant d'enfants orphelins et / ou abandonnés affectés par le VIH et le SIDA sont souvent marginalisés et à la périphérie de la pandémie du VIH et du SIDA. La conséquence est l’insuffisance de prise en compte ou d'attention accordée à l'importance du rôle qu'ils jouent dans la vie de ces enfants. L'objectif de l'étude était d'explorer comment les femmes soignantes d'enfants vivant en institution et affectés par le VIH et le sida vivent le stress lié à ces soins. Un projet de recherche qualitative avec une étude de cas a été réalisé. Les participants choisis à dessein dans un établissement de soins identifié auparavant étaient sept femmes de 35 à 59 ans. Les données ont été recueillies lors d'entretiens individuels et de discussions de groupe. L'analyse du contenu thématique des données a donné les thèmes suivants: 1) contextualiser la prestation de soins en tant que « travail»; 2) les différentes formes de stress liés à la prestation de soins; et 3) faire face et gérer le stress. Les résultats de cette étude ont indiqué que les participants étaient capables de prester des soins appropriés au sein d’une institution aussi stressante, démotivante et émotionnellement pénible. En outre, les soignants travaillant dans un environnement de VIH et de SIDA ont éprouvé un stress supplémentaire lié aux obstacles organisationnels et de gestion, au manque de soutien émotionnel et pratique, à une formation inadéquate, à des difficultés liées à la discipline exigée par ces soins et au manque de respect et d'appréciation pour les défis que rencontrent les enfants pris en charge. Il est recommandé que le soutien à la formation et à la gestion ainsi que le soutien personnalisé et les conseils aux aidants dans le contexte institutionnel pourraient les aider à mieux faire face, à se sentir appréciés et autonomes, et à élever potentiellement leur statut social au sein de la société.

## Introduction and background

In 2005, it was estimated that 2.3 million South African children would have been orphaned by the devastation of HIV and AIDS by the year 2020 (Actuarial Society of South Africa, 2005). The number of all orphaned children in the country had already reached 3.6 million in 2012, of whom two million in the age group 0–17 years had been orphaned by AIDS (UNICEF, 2013). Successful life outcomes for homeless orphaned and vulnerable children are usually dependent on factors such as the ability of members of the extended family to care for them, availability of resources, age of the child, government policy, and community-based interventions (Kuo & Operario, 2011; Mosia, 2014). Family care for orphaned and vulnerable children is usually preferred by the children and families themselves, and highly regarded by policymakers (Smart, 2003). Yet, a preferred solution may evolve into an additional complication when the responsibility for care and support falls on vulnerable guardians, including grandparents, young siblings, and relatives who may themselves be infected with HIV, thus leaving fewer available caregivers and a growing number of financially overwhelmed households (Foster, 2002; Miller, 2007).

In many countries, children in need are temporarily or permanently placed in ‘residential care’. Evidence suggests that the phenomenon of residential or institutional care has been growing in recent years due to a complex interplay of various factors, among which HIV and AIDS are predominant (Jelsma, Davids, & Ferguson, 2011; Lerner & Trivedi, 2013). South Africa is faced with the challenge of not only providing adequate institutional space for vast numbers of orphaned and vulnerable children, but also ensuring that the institution is capable of facilitating the healthy and holistic development of these children (Makiwane, Schneider, & Gopane, 2004; Morrison, 2008; Mosia, 2014).

Institutional care is mainly aimed at providing either temporary or permanent care to children for various reasons such as being orphaned, originating from a seriously unstable home and being chronically ill. In general, orphanages and child villages are the major types of institutional care models (Beard, Beard, Dimmock, & Streshley, 2001), and caregivers fulfil a crucial role in the functioning of such institutions (Mosia, 2014; Neimetz, 2011). In spite of this pivotal role, it is often found that better quality support is advocated for vulnerable children and family-system caregivers, but not necessarily for caregivers *per se* in institutional context (Adejuwon & Oki, 2011). If this deficiency is to be remedied, Jackson et al. (2004), emphasise the urgent need for pragmatic support not only for extended-family caregivers, but also for staff of institutions in order to improve care for (at least) young orphaned and vulnerable children. Still, a subtle point needs to be emphasised here: the ‘pragmatic’ support to caregivers that the authors advocate is aimed at improving service rendering to the children as ‘clients’– it may not be dedicated specifically to fostering the psychosocial well-being of the caregivers themselves. In other words, reference is made to empowering caregivers in rendering better service to others, but not to supporting caregivers as such. It may then well be asked: ‘Who will care for the carers?’

It is, however, essential to gain a better perspective on caregivers’ experience and perceptions of stress in their working world before it might be possible to consider appropriate avenues for supporting them. Consequently, this investigation was aimed at contributing by focusing on how they perceived their roles and the types of stress experienced by the caregivers of children in a formal institutional context. Unlike children raised outside of an institution, orphaned and vulnerable children are reliant on the institution as a ‘home’ environment, and therefore it follows that institutions and institutional personnel are proxies to parental and familial care (Smyke et al., 2007). This responsibility intensifies the psychosocial burden on caregivers in their duties.

## Literature review

### Caregivers and caregiving in the institutional context

As primary caretakers responsible for children’s daily needs, development, and nurturing of life experiences, caregivers in the roles of professionals or paraprofessionals are often referred to as residential workers or house parents (Kools & Kennedy, 2001). Their working activities, defined as caring work, is characterised by Davies (1995, pp. 18–19) ‘as attending physically, mentally and emotionally to the needs of another and giving a commitment to the nurturance, growth and healing of that other’. This description is germane to orphaned and vulnerable children being cared for in an institutional environment, as well as to the contextual role their caregivers fulfil. Associated with caregiving is caregivers’ stress, health risks and the risk of burden. Neimetz (2011) states that the main roles and responsibilities of caregivers are to provide protection and manage behaviour and daily routines as well as providing warmth, and closeness. Caregivers were allowed to gently discipline children just as parents would and assume additional responsibilities when another caregiver was not available to do so (Neimetz, 2011). Considering that the focus of this paper is on caregiver stress (in an institutional context), we reviewed literature specific to this context.

### The institutional context as working environment

The dearth of appropriate investigations in sub-Saharan context in this research niche necessitated the use of examples from abroad. For example, Vashchenko, Eastbrooks, and Miller (2010) in Ukraine, found that caregivers perceived their work difficulties as including conflicts, lack of cooperation, and insufficient administrative support and general work-related stress. With regard to management challenges, a lack of support and respect, perceived negativism, and lack of trust in the workplace were cited as aspects of the job that caregivers hoped would change. Others have noted that contributing factors to high staff turnover rates include dissatisfaction with low wages, high child-to-staff ratios, poor perceived professional status, and long or irregular work hours (Colton & Roberts, 2007; Evans, Bryant, Owens, & Koukos, 2004).

In Ghana, an absence of a formal system for staff training was recognised and regarded as an important need (Castillo, Sarver, Bettman, Mortensen, & Akuoko, 2012), possibly leading to caregivers’ feelings of empowerment and increasing motivation. The authors posit that through training, the personnel working in institutions might feel more empowered and motivated. Furthermore, with appropriate training, caregivers may then acquire the necessary knowledge and skills to communicate effectively with the children and respond to their physical, psychological, and emotional needs. Training of development of caregivers personal communication skills may also translate into effective inter-collegial interactions (Castillo et al., 2012). It seems that one of the most important avenues for elevating the status of professional childcare is that of improving the training, skills, and expertise of professional childcare workers (Evans et al., 2004).

Psychologically rewarding as caregiving may be considered by most of those providing it, it remains a highly demanding and stressful job or commitment (Ohaeri, 2003; Pavalko & Woodbury, 2000), and caregivers require appropriate psychosocial support in the functions that they fulfil.

### Experiences of caregivers

Working in the caregiving profession increases the likelihood of adverse psychological outcomes for caregivers (Figley, 2002; Sabin-Farrell & Turpin, 2003). Stressors have been linked with negative mental health outcomes such as depression and anxiety (Ellison, 1995). In particular, caregivers in AIDS care centres in general (i.e. not only for children) were prone to high levels of stress because of their work (Hayden & Otaala, 2005). Where caring for orphaned and vulnerable children is concerned, the demands on caregivers would be higher, given the need for increased sensitivity to the children’s nurturing needs. Research in this area of caregiving suggests that caregivers are subject to a sense of bearing an extra or heavier responsibility because of the physical and psychological costs of caring for children affected by HIV and AIDS (Hayden & Otaala, 2005; O'Neill & McKinney, 2003). A study conducted in five countries, including South Africa by Razavi and Staab (2010) established that most caregivers care for children who are often sick or have psychological difficulties; thereby experiencing high levels of stress because of work overload and lack of adequate support from the institutions that employ them. A further source of caregiver stress derives from the fact that they have had little or no access to training opportunities thereby being unable to enhance their skills and address the overwhelming nature of their work demands, resulting in exposure to high levels of burnout, stress, lack of motivation and job performance difficulties (Razavi & Staab, 2010).

Specifically in the milieu of caregiving for orphans and vulnerable children, caregiver stress can be divided into primary and secondary components. Primary stress arises from having to care directly for these children and is experienced because of everyday caregiving duties such as assisting the children with bathing, toileting, managing behaviour, and planning of daily care. Secondary stressors are indirectly related to caregiving and comprise caregiver conflict with relatives of the children, exposure to the economic hardship suffered by the children and their families, as well as caregivers’ personal experience and limitations on their own family life, leisure, and social activities (Pearlin & Aneshensel, 1994; Primo, 2007).

Curbow, Spratt, Unagaretti, McDonnel, and Breckler (2000) describe work-related stress as a predictor of burnout, internal conflicts, high staff turnover, and dissatisfaction. Evans et al. (2004) state that prolonged exposure to chronically unstable and stressful work conditions leaves childcare professionals vulnerable to burnout. Because of society’s ‘unrealistic expectations’ of caregivers (Rowe, 2003, p. 17), a considerable amount of stress is placed on them, compromising their health both physically and psychologically. This may lead to decreased psychological health and life satisfaction, and increased levels of caregiver burden, role strain, and depression (Figley, 2002; Fredriksen-Goldsen, 2007; Sabin-Farrell & Turpin, 2003).

### Burnout and caregiver burden

The term ‘caregiver burden’ describes the physical, emotional, financial, and social problems associated with caregiving (Given et al., 2004; O'Neill & McKinney, 2003), which in turn may lead to burnout, a multidimensional syndrome that is characterised by emotional exhaustion, depersonalisation, and reduced personal accomplishment. Emotional exhaustion, the basic stress dimension of burnout, entails malaise and loss of energy leading to feelings that emotional resources are inadequate for providing care to others. Depersonalisation, the interpersonal dimension of burnout, is the negative attitude of dehumanising perception whereby caregivers detach themselves from those under their care and manifest cynicism, apathy, and withdrawal. The final component, lack of personal accomplishment, leads caregivers to feel that they are incompetent and cause them to perceive themselves as unable to reach work-related goals and as incapable of achieving in the workplace. This is evident in decreased efficiency at work, difficulty in concentrating, and increased irritation with colleagues (Evans et al., 2004; Lakin, Leon, & Miller, 2008).

The concept of caregiver burden furthermore relates to resources that are used and pressures created by working in an environment of caring. The burdens are predictors of the measure of stress that caregivers themselves perceive to undergo. The assumption is that if caregivers have fewer caregiver burdens, they would also experience less caregiver stress (DiBartolo & Soeken, 2003; Pruchno, Kleban, Michaels, & Dempsey, 1990). Low levels of personal accomplishment are also factors associated with a heightened sense of caregiver burden (Evans et al., 2004). The suggestion is that experiencing low personal accomplishment may be the most common component of burnout and that childcare professionals in particular are vulnerable to this element (Evans et al., 2004; Razavi & Staab, 2010; Sears, Urizar, & Evans, 2000).

Caregiver stress may manifest itself in various ways. For example, in Kenya, research by Mutiso, Chesire, Kemboi, Kipchirchir, and Ochieng (2011) found that caregivers experienced frequent headaches because of long working hours, work pressure, tension among staff, and multiple directives from senior staff members. Memory problems, anxiety, depression, and aggression are equally some of the effects of stress on child caregivers, and the resultant high staff turnover in itself may add to stressor factors (e.g. training and assisting new members). Further deleterious effects in child caregiving environments may include absenteeism, heart disease, high blood pressure, and substance abuse. High emotional involvement without adequate social support or feelings of personal work accomplishment (e.g. job satisfaction) may leave caring professionals vulnerable to job burnout (Adams, Boscarino, & Figley, 2006; Hlabyago & Ogunbanjo, 2009; Razavi & Staab, 2010). It may therefore be assumed that if caregiving staff is adversely affected, the care and support of children in children’s homes will suffer because of a decrease in quality of service (Mutiso et al., 2011).

In light of the literature reviewed, the main questions that guided this paper were:

- What are the stresses linked to caregiving in this particular context?
- How do caregivers cope with stressful experiences arising in their work environment?

## Methods

We conducted this study within an interpretivist paradigm following a qualitative approach and case study design. This approach allowed us to conduct research with caregivers in their accustomed working environment in order to explore and understand their subjective experiences.

### Study participants and setting

This study aligned with one of the findings of a broad study indicating that the well-being of institutionalised HIV and AIDS affected children was largely dependent upon the quality of caregiving experiences (Mohangi, 2008). The current study aligns with the second track of data collection and explores how stress affects caregivers’ caregiving practices in this residential care facility at a major provincial hospital situated on the outskirts of Pretoria. This care facility is home to orphaned and vulnerable children affected by poverty, abandonment, and abuse, but predominantly by HIV and AIDS. The government aided care facility cares for 35 male and female children below the age of 10 years. Fifteen caregivers, assisted by support staff, are employed at this home.

The participating caregivers, purposively selected because of their role as caregivers in the institution, were females in the age range of 35–59 (Table 1). From among the seven participants, four were purposively reselected for individual interviews because of their fluency in English, rich verbal participation in the focus group discussion, and experience as caregivers.

**Table 1.** Participants’ details.

Participant (all female)	Age group					Working experience at institution (years)
	20–29	30–39	40–49	50–59	60–69	
Caregiver 1			✓			3
Caregiver 2			✓			11
Caregiver 3		✓				2
Caregiver 4			✓			5
Caregiver 5			✓			4
Caregiver 6				✓		11
Caregiver 7			✓			11

## Researchers’ roles

To guard against biases, we strived to counter such influence through reflexivity. Our researcher journal served as a valuable instrument for recording thoughts, which allowed for critical reflection. Furthermore, we strived to ensure that our race, gender and social standing did not influence our researcher roles.

In this study, member checking was addressed during the semi-structured interviews by the facilitators of the focus groups and the interviewers through continually verifying and summarising the participants’ remarks. This provided the participants with an opportunity to clarify any misconceptions with regard to their statements. Such approaches ensured our attempt to mitigate against bias and/or errors.

### Data collection

Data collection took place on the premises of the selected facility, and two data collection strategies were employed. Firstly, a two-hour focus group discussion was conducted with the seven participants in order to listen to their general perspectives and to ease them into the process (Maree, 2007). Such interaction among the participants according to Kvale (1996, p. 101) ‘often leads to spontaneous and emotional statements about the topic being discussed’. Open-ended questions were asked and the discussion was moderated in order to gather information. Although all the participants were able to communicate in English, an interpreter was also used to aid comprehensibility.

Secondly, the individual semi-structured interviews conducted with four participants to explore caregivers’ experiences at a deeper level, were guided by open-ended questions focusing on the participants’ points of view, perceptions, and beliefs about caregiving, their job satisfaction and stress. Participants were purposefully selected based on their ability to communicate in English, their rich verbal participation in the focus group discussion, and their experience and involvement as caregivers. All discussions and interviews were audio-recorded to facilitate careful and accurate capturing of participants’ responses.

## Ethical considerations

Standard ethical procedures were maintained, with particular sensitivity to issues of confidentiality and anonymity. Ethical clearance was obtained through the overseeing university’s research and ethics committees. Accurate and complete information was conveyed to participants in order for them to fully comprehend the nature of the research and consequently make a voluntary and reasoned decision regarding their participation in the study. Participants were informed of their right to withdraw at any stage with no consequences. Special care was taken to ensure that the participants did not feel coerced into participating. Explanations of the processes were provided to participants in their mother tongue via an interpreter. The participants were furthermore given the opportunity to ask for clarification of any matters not entirely clear to them. The caregivers subsequently provided their written informed consent to participate in the study.

## Data processing and analysis

The audio-recordings were translated from Sesotho to English where necessary and transcribed to facilitate scrutiny and analysis (Kvale, 1996). A Sesotho-speaking psychology student conducted the translations. The researchers without discrepancy jointly conducted the analysis of the data. During data analysis, we focused on the participants’ subjective experiences and perceptions, as well as their understanding of stress. Accordingly, key words, meanings, and themes emerged as important to the description of the phenomenon. Braun and Clarke’s (2006) method was used for thematic content analysis, with the first phase entailing familiarisation with the data and noting of initial ideas. The second phase constituted generating initial codes by coding significant aspects of the data systematically. In the third phase, codes were grouped into potential themes, which were then reviewed during the fourth phase to ensure that they correlated with the coded extracts and the entire data set. The fifth phase allowed for ongoing analysis in order to refine the particulars of each theme. We ensured that the thematic content analysis be reported in a valid and understandable manner (Elo et al., 2014).

## Results and discussion

The initial themes arising from the analysis of the raw data were clustered into three main groups with their related subthemes: (1) contextualising caregiving as ‘work’, (2) stresses linked to caregiving and (3) coping with stress.

### Contextualising caregiving as ‘work’

This theme was clustered from patterns in the data related to placement or localisation in a caregiving environment; in other words, the reasons provided by participants for becoming involved in this particular working context or choosing it as a profession. This theme, furthermore, explores how participants construct their role of caregiving in an institution for orphaned and vulnerable children.

The participants indicated that they began working as caregivers because of a love for children and an innate ability to care for them. It was clear to the researchers that the caregivers were initially motivated by a strong altruistic impulse and felt themselves driven by an awareness of what they considered to be a talent: *I have this ability*; *potential to work with kids; It’s because if you love kids, that’s why you come to work. There are so many* [*other jobs*]*, which we can do, but because you love kids we are here*. Caregivers at this institution seemed to have formed a vision of dedication to vulnerable children: *You see, if you do not love the child you cannot stay for five years, six years* [*will not last so long in this type of employment*]*, but some of us stayed here for five years*. However, despite this dedication towards their job, a few of the caregivers expressed a loss of dedication and disillusionment with their work, feeling trapped in their jobs and caregiving role because of a need to care for their own families while lacking alternative job opportunities: *It* [*doing caregiving work*] *ends up not being with love now; we just sacrifice ourselves to come and work. That love* [*dedication we used to possess*] *is no longer in us now; it is just because we have something* [*a job*] *to do.* From the caregivers’ responses, it could be inferred that it was not so much the lack of *love for the kids* but the stressors and associated burnout that they felt despondent, apathetic, indifferent, and uninterested: *I am just working for the sake of working*.

In terms of their role, the participants tended to take a strong practical view of their duties and routine daily caregiving activities such as hygiene, nutrition, medical care, and educational care. *Taking care of them* [*children*]*,* [*involves*] *cleaning them, feeding them, taking them to the hospital, to doctors, giving them the medication, cooking for them during the weekends, prepare uniforms for them, and lunch boxes. We must teach them* [*help with their*] *homework and sometimes we read Bible stories to them*.

Since such ‘quotidian’ activities are likely to predominate in an institutional setting, and particularly in view of the considerable numbers of children involved, it is possible that the caregivers’ first thoughts about their role would focus on practical daily matters. Yet, other responses indicated that they were aware of the linkage between quotidian activities and the more subtle aspects of a parenting role; in other words, they also perceived and defined their role construct on the same tenets as that of a parental figure. *I am a mother: I have children, and these are children. I treat them like my own children.* It seems that while the caregivers know that they are employed to care for children and they regard this as their ‘work’, their innate love for children appears to give them a semblance of satisfaction.

### Stresses linked to caregiving

It may be expected that individual caregivers will perceive their working environment in an individual way and consequently experience stress in a unique manner. However, it may also be expected that common salient points will be identifiable in these experiences and perceptions. This second theme incorporates the experience of stress in relation to caregiving practices in the institutional context.

Central to the stress experiences of the caregivers were the institution’s management aspects, maintaining discipline among the children, and a lack of authority as (pseudo-) parental figure. Especially regarding their relationship with the management of the institution, the participants considered the following to be stress inducing:

- lack of adequate support by management (a lack of guidance, advice, and training): *I can’t say we* [*are*] *getting the support from management. We do not have any training*.
- poor communication (one-sided communication from management and lack of true dialogue with caregivers in proper meetings and debriefings): *If you talk, they say they don’t want to hear about that*; *We are not allowed to talk. I think if the management can listen sometimes to what we want from them, then everything can be all right*.
- an authoritarian and even threatening management style (giving rise to a perception of victimisation among caregivers): *We don’t discuss the issues, they tell us* [*merely inform us about them*]*. The board members, they do not even come* [*to see us*]. *If you strike, you are fired*.
- management’s tendency to leave staff to their own devices, placing them in a quandary by depriving them of the right and means to formulate and implement their own solutions to daily problems (thus smothering any positive initiative among caregivers): *The social worker will tell you to do whatever is best for you. On the other hand I must do what suits me,* [*yet*] *if I do something wrong to these kids, I am the one to be blamed. Therefore, you see, I am in between two things*.
- loss of inner drive and personal motivation among caregivers (giving rise to despondency, purposelessness, and depersonal­isation)); *We are no longer enjoying it* [*our careers*]*; At first I do* [*did*] *enjoy working here, but now, things are becoming difficult. If I can get the opportunity to quit, I am going to use it*.
- inadequate remuneration and benefits for staff: *It is not all right for me or for the years I’ve worked here and for the work I’m doing. I do not earn not even three thousand rand*.
- There were strong indications of a need for training for caregivers especially in maintaining discipline (i.e. orderliness and good behaviour) among the children more effectively. Caregivers appeared burdened with the responsibility of maintaining discipline among the children, but were not equipped with either the necessary approaches to do so or the required support from management. *They* [*management*] *said we are* [*acting as*] *the parents; see to it that you discipline them* [*the children*] *in which way* [*you see fit*]*. Do not wait for a social worker. So, but if you can discipline them, they* [*the children*] *say you are abusing them.* The stress that they experienced in such cases resulted in a withdrawal response, which culminated in a persistent feeling of indifference to their work and the children.

Caregivers also stated that they do not receive any training in caring for the children and in managing their behaviour. They are largely left to find their own resources: *It is for my own baby* [*up to me*] *to go see what can I do. She* [*social worker*] *offers one hour* [*of guidance and advice*]*. I think it is not enough*.

An exacerbating factor was the apparent lack of respect for caregivers in their parental or pseudo-parental role, particularly among the older children. This engendered in caregivers feelings of being helpless, unwanted, and unappreciated, especially in having their efforts and sacrifices thrown back at them in disrespect and rejection through the hurtful phrase ‘[*you’re*] *not my mother’.* The affective injury that caregivers experienced in the rebuff of their attempts to fulfil a ‘*mothering’ or ‘homemaking*’ role was compounded by the insult of being regarded by the children as mere ‘*paid cleaners’.* It can be concluded that such stress would in all likelihood only add to feelings of despondency and emotional blunting.

### Coping with stress

This theme considers how caregivers cope with the challenges they encounter in working with the HIV and AIDS affected children in the institutional setting. Coping is defined as ‘the cognitive and behavioural efforts made to master, tolerate or reduce external and internal demands and conflicts among them’ (Lazarus & Folkman, 1984).The caregivers reported coping in three major ways with the stress that they experienced. Firstly, religious beliefs were pointed out as a major coping strategy. Secondly, participants highlighted the importance of the support that they received from fellow-caregivers at work and from their family members at home. Thirdly, caregivers utilised a range of personal coping strategies.

Firstly, religious beliefs were pointed out as a major coping strategy. Personal faith and beliefs as a deep-seated inner resource constituted a supportive element for the caregivers by allowing them to draw on what they considered ‘*God’s grace’* in order to attain a sense of meaning and hope. *Truly speaking, it is God’s grace because after a kid swears onto* [*at*] *you and then you go back to her and* [*in a*] *loving* [*manner*]*, you know how difficult is it? It is difficult, but I just told myself that it is really God’s grace to do all of those things to accommodate them.* This means of coping seemed to focus on two elements: asking for energy to deal with difficulties, and asking for the power to exert self-control over feelings of aggression. When caregivers were struggling to find meaning in what they were doing, or were faced with challenges, they would turn to prayer to help them find strength and counter the stress that they experienced. *I am just praying, God must give me strength to come to work and communicate with the children. Give me love and strength to work with them’*.

Secondly, the support that caregivers received from their family members at home and fellow-caregivers at work was highly significant to them in managing challenges. At home, by sharing stressful experiences of the day with family members, caregivers found a way of mediating stress through having someone outside the working environment listen and provide understanding. At work, caregivers found great value in self-initialised seeking of peer support, and they appeared to support each other by allowing their colleagues to take time off when they felt stressed and by taking on an extra burden on a colleague’s behalf. In interacting with their peers, and by sharing their experiences and challenges in caregiving, the caregivers ultimately also shared coping strategies in dealing with similar circumstances and types of stressors: *I speak to my colleague, ‘You know, today he* [*a child*] *was doing this and I don’t like the way he act* [*and I didn’t approve of the way he acted*]*. We talk* [*discuss things among ourselves*]*. Sometimes you feel like we have a shoulder to cry on*.

Thirdly, the findings indicated that caregivers utilised a restricted range of personal coping strategies. Whereas many of the caregivers appeared to avail themselves of a social support system (e.g. family and colleagues), the majority made use of passive and emotive coping when experiencing a sense of helplessness. Thus, the predominant coping mechanisms to which they took recourse in such cases appeared to be withdrawal and isolation through sleeping or physically leaving the immediate work environment, emotional palliation through crying, deciding to deal with problems on their own terms and according to their own lights (whether effective and appropriate or not), and emotional indifference to excessive or unfair demands from others*: So you do your job. You go to your room and you cry, you satisfy yourself that you let all out, do whatever is needed for that day. That helps you to release some of the stress you are feeling. You isolate yourself, you cry, you give off your emotion, and then you are okay*.

Despite some attempts to reach out for support from others, it can be concluded that, in the main, caregivers internalised the stress they experienced in order to cope and would rather utilise emotion-focused responses such as behavioural escape-avoidance and distancing as identified by Folkman, Chesney, and Christopher-Richards (1994). A particularly significant approach that some participants in this study strongly hinted at, and which can be subsumed under the subtheme of ‘personal strategies’, was the eminently ‘pragmatic’ one of withdrawing from the field of stress by seeking other employment. A measure of irony is attached to this strategy, since it could only be commented on fully by those who had already used it and, consequently, were not present anymore. Although participants mentioned that new employment was not easy to find and that they were reluctant to leave their current work, stressful as they might experience it, they noted that the average period of remaining at the institution was approximately five years.

## Discussion

The findings from this study suggest that the caregivers experience an imbalance between the resources, capacities, and managerial support available to them on the one hand, and the demands imposed upon them by their working role and environment on the other. The persistent demands on caregivers in an institution were shown in this study as causing strain on their coping resources and problem-solving capacities (Cohen & Wills, 1985). The caregivers in this study appeared to exhibit high levels of stress, as well as burnout associated with their experiences. They used a variety of coping strategies that included problem-focused coping (seeking social support) to deal directly with challenges, but for the most part took recourse to dysfunctional coping and avoidance, as reflected preponderantly in refraining from taking any action in difficult situations.

Caregivers are considered the primary caretakers of the children in their care by attending to their physical, mental, and emotional needs (Davies, 1995). There are contextual and environmental factors that influence the conception of caregiving (Leira, 1994). The formal caregiver views caring within this concept as ‘work’ (a job) and thus is depicted as significantly more focussed upon the activities conducted, than the emotional connection (Uren, 2009). During the interviews conducted for this study, it became evident that the caregivers in the institution concerned fulfilled a role beyond the limits of routine, practical or *instrumental* caregiving, but also assumed what Mohangi (2008) refers to as a *pseudo-parental role* and require the same kind of care as those living in families (Fyhr, 2001; Neimetz, 2011). The institution then becomes an ‘artificial family institution’ where caregivers fulfil the parental role thereby ‘providing physical care and a psychological parent-child relationship as well as a model of morally sound behaviour’ (Fyhr, 2001, p. 62).

Lazarus and Folkman's (1984) assertion that a person's perception of how much personal control she or he has, is of significance in moderating the effects of stress and facilitating coping. The need for training of caregivers appears to be paramount (Castillo et al., 2012; Colton & Roberts, 2007; Evans et al., 2004; Hlabyago & Ogunbanjo, 2009; Jackson et al., 2004; Van Dyk, 2008). The current study highlights in particular the need for training in behaviour management in order for the caregivers to discipline and respond effectively to the children in their care.

Knowledge would provide confidence within their position and identity as a caregiver as well as competence in their abilities to rather than a sense of helplessness (Scott & Brown, 2004). Lack of necessary caregiving support and training results in confusion and distress among caregivers and demoralises them in the performance of their duties (Hlabyago & Ogunbanjo, 2009). If exposed to the appropriate training, caregivers’ appraisal (Lazarus & Folkman, 1984) of the challenge of behaviour management should alter positively and they should be able to apply appropriate problem-solving strategies and, consequently, avail themselves of problem-focused coping. Simultaneously, appropriate training may assist them in improving their communication with colleagues and administrators within the institutional setting (Castillo et al., 2012).

Early researchers such as Cohen and Wills (1985) and Folkman and Lazarus (1985) noted that the ability of caregivers to manage stressful situations were influenced by their experience of their particular situation as demanding or challenging. If caregivers feel that their health and energy are not at an optimal level, it makes it more difficult for them to cope effectively with stressors both primary and secondary resulting from the demands of their work (Lazarus & Folkman, 1984). The findings from these authors may be applied to the case of the caregivers in the current study. The primary source of stressors, as identified by Pearlin and Aneshensel (1994), can be found. The findings in this category correlate significantly with the strategy proposed by Van Dyk (2008) in preventing occupational stress and burnout among HIV and AIDS caregivers:

- a supportive working environment;
- professional supervision and mentoring;
- emotional support and therapeutic counselling;
- stress reduction and coping skills;
- ongoing training.

Van Dyk (2008) states that both caregivers and management should work as a team in order to address HIV and AIDS effectively in their interventions. This necessity was underscored by the responses obtained from participants during the current study, considering the problems that arise when communication between management and caregivers is poor in the day-to-day care tasks and activities in which caregivers are engaged. If provided with advice, training and guidance the caregiver may be able to utilise it as a personal resource that they would be able to rely upon for support and at times a sense of relief (Kramer, 1993) as well as buffering the effects of stress within caregiving and the institution through effective problem-focused coping.

As can be seen from the responses of the participants in this study, religion or spirituality can be viewed as a strong source of support for the caregivers in providing them – as noted by researchers such as Büssing, Fischer, Ostermann, and Matthiessen (2008) – with a sense of strength, reassurance, comfort, and hope in distressful circumstances. Social support is defined as ‘helpful functions performed by significant others such as family members, friends, co-workers and neighbours and thereby enhances the individual’s physical and psychological well-being’ (Throits as cited in Petersen, 2000, p. 10). This is well illustrated in the responses from participants in this study, but with the proviso that a balance should be maintained between sharing and burdening.

## Conclusion

Findings from this study indicated that participants experienced caregiving in an institution as stressful, demotivating, and emotionally burdensome. Moreover, caregivers working in an environment of HIV and AIDS were constantly experiencing additional stress and challenges related to organisational and management impediments, especially lack of emotional and practical support, inadequate training, discipline difficulties, and lack of respect and appreciation from the children in their care. It seems that they stand on the periphery of the devastating effects of the pandemic and have yet to be recognised for the multiple altruistic roles they play. It further appeared that these stress and challenges had begun to overwhelm the caregivers, leaving them feeling exhausted, and with a sense of depersonalisation and reduced personal accomplishment. It can therefore be inferred that the caregivers who participated in this research were experiencing burnout, or are at least displaying significant signs of it, as described in various studies on the subject (e.g. Curbow et al., 2000; O'Neill & McKinney, 2003).

While caregiving in any environment evokes images of stress and emotional strain, it is difficult to comprehend the full psychosocial impact of providing daily care to orphaned and vulnerable children primarily affected by HIV and AIDS in a residential care environment. Insights from this study emphasise the necessity of adequate support on multiple levels required by caregivers. Given caregivers’ experiences of what constitutes stress, it seems plausible to assume that stress management skills could possibly assist them in coping with their daily tasks.

We thus argue that a great need exists for ongoing training programmes that teach effective coping strategies to caregivers who find themselves overwhelmed by the challenges of their jobs. It is equally essential that opportunities be granted to them for regular ‘debriefing’ and personal guidance counselling in which their physical and psychological well-being is the primary focus. Such initiatives in self-care and self-awareness skills training may contribute significantly to the prevention of burnout. Considering the effects of management challenges on caregiver stress, we recommend additional research specifically focused on this area.

It is also recommended that managers of HIV and AIDS care institutions should be in constant interaction with the caregivers in order to avoid miscommunication and feelings of discontent or depersonalisation that might eventually lead to burnout. Caregivers should be given the opportunity to voice their concerns and be consulted frequently to identify their needs and address them adequately. Future research could further assess formal caregivers’ access to coping resources and interventions within an institutional environment, as well as undertake evaluations of the effectiveness of strategies for managing various work-related stressors.

Further investigations are also needed to elucidate how care challenges within an institutional setting impact upon carer well-being. This could be expanded to encompass the familial context of caregivers’ lives, considering the importance that several participants assigned to family life in their emotional well-being and handling of stress.

A particularly pertinent issue is that of research that could be conducted into interventions to help caregivers identify the appropriate fit between coping strategies and specific sources of stress in order to assist them in the management of occupational stress.
